# Pretreatment neutrophil-lymphocyte ratio and platelet-lymphocyte ratio as prognostic biomarkers for neuroblastoma risk stratification

**DOI:** 10.3389/fimmu.2026.1831234

**Published:** 2026-06-15

**Authors:** Lixiao Shi, Dixiao Zhong, Ruihong Tang, Duanfang Shao, Xiang Cheng, Zhaoxia Zhang, Rong Liu

**Affiliations:** Department of Hematology, Capital Center for Children’s Health, Capital Medical University, Beijing, China

**Keywords:** neuroblastoma, neutrophil-to-lymphocyte ratio (NLR), pediatric oncology, platelet-to-lymphocyte ratio (PLR), prognostic biomarkers, risk stratification

## Abstract

**Background:**

Neuroblastoma (NB) is the most common extracranial solid tumor in children, accounting for approximately 15% of pediatric oncology mortality. While risk-stratified therapy has improved survival, high-risk cases still face poor outcomes. Pretreatment inflammatory markers, including the neutrophil-lymphocyte ratio (NLR) and platelet-lymphocyte ratio (PLR), have emerged as potential, cost-effective prognostic biomarkers to refine risk assessment and guide treatment intensity.

**Objectives:**

This study aimed to investigate the associations between pretreatment NLR, PLR, and clinicopathological characteristics, and to evaluate their significance in neuroblastoma risk stratification.

**Methods:**

We conducted a retrospective study of pediatric patients newly diagnosed with neuroblastoma at the Capital Center for Children’s Health, Capital Medical University, between March 2023 and July 2025. Data, including age, International Neuroblastoma Staging System (INSS) classification, and International Neuroblastoma Risk Group Staging System (INRGSS) criteria, were recorded. Pretreatment blood samples were analyzed for neutrophil, lymphocyte, and platelet counts to calculate NLR and PLR. The association between these ratios, clinicopathological characteristics, and risk groups was analyzed using Receiver Operating Characteristic (ROC) curves.

**Results:**

ROC curve analysis established optimal cutoff values for NLR and PLR at 0.98 and 104.6, respectively, for differentiating high-risk from low/intermediate-risk patients. The combined NLR-PLR model demonstrated superior predictive performance, yielding an Area Under the Curve (AUC) of 0.833. Both NLR and PLR showed significant positive correlations with neuroblastoma risk stratification. Notably, patients with elevated baseline NLR and PLR values were significantly more likely to present with advanced disease stages compared to their lower-risk counterparts. These data suggest that the integration of NLR and PLR into a dual-index score synergistically enhances the accuracy of risk discrimination.

**Conclusions:**

Pretreatment NLR and PLR are potent, cost-effective, and easily accessible biomarkers that correlate significantly with high-risk neuroblastoma and poor prognostic indicators in unadjusted analyses. Incorporating these inflammatory indices into standard staging protocols may improve the precision of initial risk stratification, allowing for more personalized therapeutic interventions.

## 1 Introduction

Neuroblastoma (NB) is a highly heterogeneous extracranial solid tumor originating from sympathetic nervous system progenitor cells of the neural crest lineage (1, 2). Most frequently presenting as adrenal or abdominal lesions, NB accounts for approximately 7–8% of all childhood malignancies but disproportionately contributes to nearly 15% of pediatric cancer-related mortality (3, 4). The disease is characterized by a strikingly diverse clinical course, ranging from spontaneous regression in certain biological subtypes to aggressive, rapid metastatic progression in others.

Current risk stratification categorizes patients into very low-, low-, intermediate-, and high-risk groups. While survival rates for low- and intermediate-risk cohorts are excellent, the 5-year survival for high-risk patients remains below 50% despite intensive multimodal therapy (4, 5). Given this disparity, there is an urgent clinical imperative to identify novel, minimally invasive, and feasible biomarkers that can enhance risk stratification at the time of diagnosis.

Systemic inflammation is a hallmark of the tumor microenvironment (TME), playing a pivotal role in carcinogenesis, tumor staging, and disease progression (6). Specifically, hematological indices such as the Neutrophil-to-Lymphocyte Ratio (NLR) and Platelet-to-Lymphocyte Ratio (PLR) have emerged as robust indicators of systemic immune status. While these biomarkers have demonstrated significant prognostic value across various adult and pediatric malignancies—including gastric, lung, and Wilms’ tumors—their utility in neuroblastoma remains under-explored (7–16).

This retrospective study aims to evaluate the correlation between pretreatment NLR/PLR and clinical risk stages in neuroblastoma. By investigating these ratios, we seek to establish NLR and PLR as independent, cost-effective prognostic biomarkers to refine risk stratification and guide clinical decision-making.

## 2 Materials and methods

### 2.1 Study population and patient selection

This single-center retrospective cohort study evaluated 38 consecutive, treatment-naive pediatric patients diagnosed with neuroblastoma (NB) at the Capital Center for Children’s Health, Capital Medical University, between March 2023 and July 2025.

**Figure 1:**
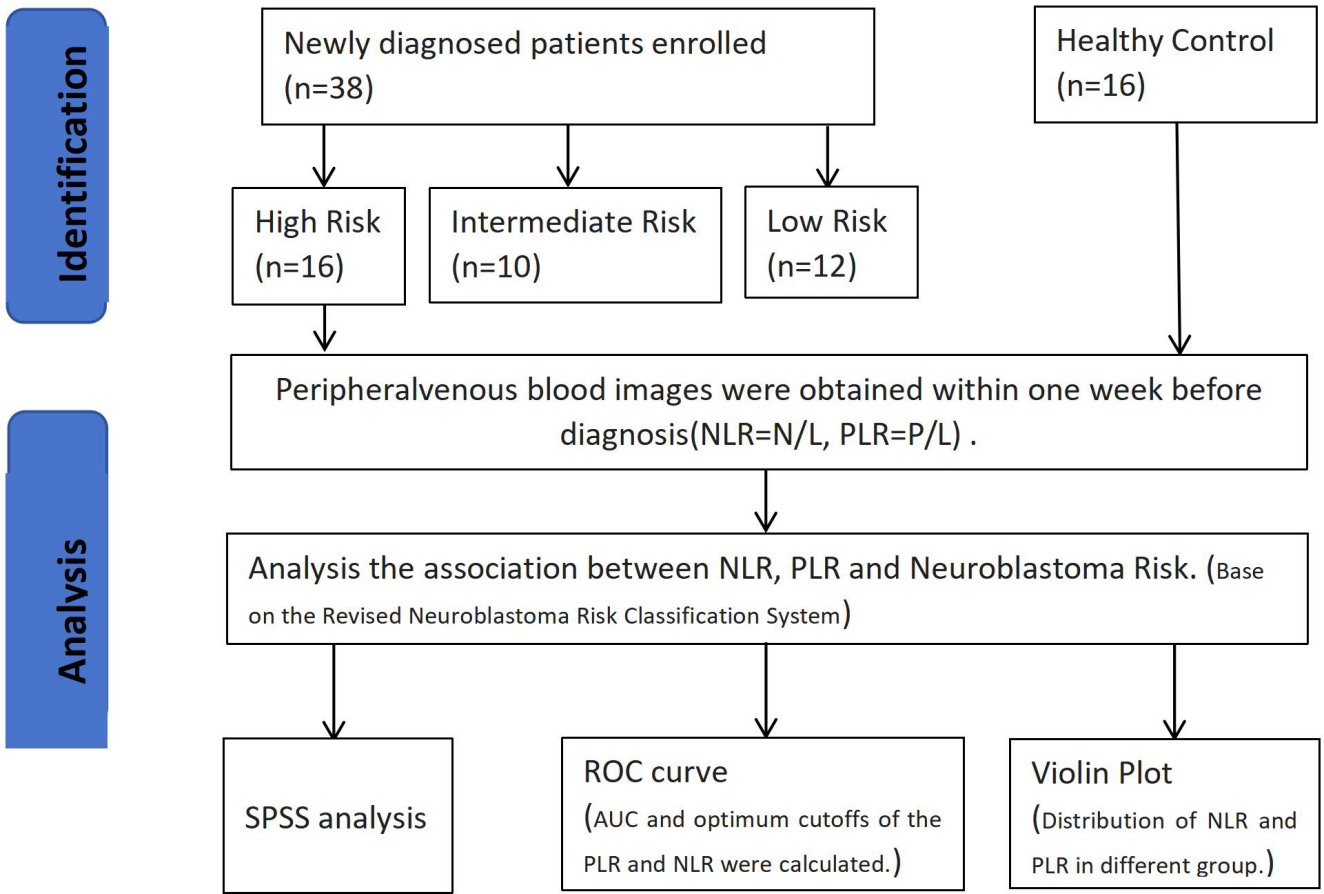
The patient screening process and study design flow diagram.

Eligible participants met the following inclusion criteria:

1. Comprehensive diagnostic documentation: Confirmed histopathological categorization (International Neuroblastoma Pathology Classification), International Neuroblastoma Staging System (INSS) staging, and International Neuroblastoma Risk Group Staging System (INRGSS) stratification.
2. Molecular and cytogenetic profiling: Central reference laboratory data including MYCN amplification status (via FISH), DNA index (via flow cytometry), and segmental chromosomal aberrations (1p deletion or 11q loss via SNP-array).
3. Complete baseline records: Availability of full pretreatment laboratory results and peripheral venous blood counts obtained within seven days prior to diagnostic intervention or therapy.
4. Treatment status: No history of prior oncologic therapy.
  Exclusion criteria included an active infectious process, a documented persistent inflammatory state, or evidence of overt bone marrow involvement (defined as bicytopenia or pancytopenia on a complete blood count). For comparative analysis, a control cohort of 16 healthy children (hematopoietic stem cell transplantation donors) was enrolled during the same period. The study design and screening workflow are detailed in **Figure 1**. Ethical approval was granted by the Institutional Review Board of the Capital Center for Children’s Health, and written informed consent was obtained from all legal guardians.

### 2.2 Data acquisition and biomarker calculation

Demographic data, clinical laboratory results, radiographic findings, and histopathological reports were systematically extracted from electronic medical records. Absolute counts for neutrophils (N), lymphocytes (L), and platelets (P) were recorded from baseline blood samples. The systemic inflammatory indices were calculated as follows:

NLR: Absolute Neutrophil Count/Absolute Lymphocyte Count.

PLR: Absolute Platelet Count/Absolute Lymphocyte Count.

### 2.3 Statistical analyses

Descriptive statistics were utilized to characterize the cohort, with categorical variables expressed as frequencies and percentages. The correlation between inflammatory indices and neuroblastoma risk levels was analyzed for significance. Optimal threshold values (cutoffs) for NLR and PLR in predicting risk stratification were determined using Receiver Operating Characteristic (ROC) curve analysis. The diagnostic performance was quantified by calculating the Area Under the Curve (AUC), alongside corresponding sensitivity and specificity. To further reinforce the robustness and clinical value of the model, we conducted internal validation using 1000 bootstrap resamples. In addition, we performed calibration analysis with the Hosmer–Lemeshow test, decision curve (DCA) analysis, and confusion matrix analysis reporting sensitivity, specificity, AUC and F1 score. Statistical significance was established at a two-tailed P < 0.05. All analyses were performed using SPSS version 24.0 (IBM Corp, Armonk, NY).

## 3 Results

### 3.1 Clinicopathological features of patients with neuroblastoma and healthy control

In accordance with the study protocol, 54 children were enrolled, comprising a neuroblastoma cohort (n=38) and a healthy control group (n=16). The neuroblastoma cohort was further stratified by risk: low-risk (n=12), intermediate-risk (n=10), and high-risk (n=16).

Baseline demographic and hematological profiles are summarized in **Table 1**. Notably, we observed a distinct trend in systemic inflammatory markers across the risk groups. High-risk patients exhibited a markedly lower median Absolute Lymphocyte Count (ALC) of 1.87 × 10^^9^/L compared to 4.5× 10^^9^/L in the low-risk group. Consequently, median NLR and PLR values were highest in the high-risk cohort (1.31 and 143.2, respectively), reflecting a more pronounced systemic inflammatory state in advanced disease stages.

**Table 1:** Characteristics of neuroblastoma patients and healthy controls.

Variables	Low-risk	Intermediate-risk	High-risk	Controls
Number	12	10	16	16
Age	2Y4M	2Y7M	3Y1M	7Y
Median (range)	(10M-4Y)	(3M-9Y)	(1M-5Y)	(10M-4Y)
Gender				
Male	4	2	13	6
Female	8	8	3	10
ANC^a^	3.0	3.12	2.43	3.43
Median (range)	(1.55-5.02)	(1.15-5.21)	(0.97-7.98)	(2.14-5.77)
ALC^b^	4.5	3.94	1.87	2.82
Median (range)	(2.89-9.81)	(1.07-6.29)	(0.7-5.83)	(2.97-4.18)
PLT^c^	369.4	401	301	318.5
Median (range)	(115-536)	(234-588)	(124-582)	(209-431)
NLR^d^	0.73	0.81	1.27	1.16
Median (range)	(0.32-1.39)	(0.2-2.71)	(0.43-10.22)	(0.74-2.0)
PLR^e^	88.0	97.2	137.8	103.6
Median (range)	(35.8-147)	(55.1-224.2)	(34.1-555)	(69.9-162)
Fer	37.17	74.55	271.89	
Median (range)	(17.6-186.6)	(35.2–440.9)	(31.7-1827.1)	--
LDH	255.5	235	491	
Median (range)	(198–434)	(190–394)	(283–3370)	--

^a^ ANC, absolute neutrophil count, ×10^9/L;.

^b^ ALC, absolute lymphocyte count, ×10^9/L;

^c^ PLT, blood platelet count, ×10^9/L;

^d^ NLR, neutrophil-lymphocyte ratio;

^e^ PLR, platelet-lymphocyte ratio;

^f^ Fer, ferritin, ng/ml;

^g^ LDH, lactate dehydrogenase, U/L.

### 3.2 Determination of optimal NLR and PLR cutoff values

#### 3.2.1 Discriminatory capacity and ROC analysis

**Figure 2:**
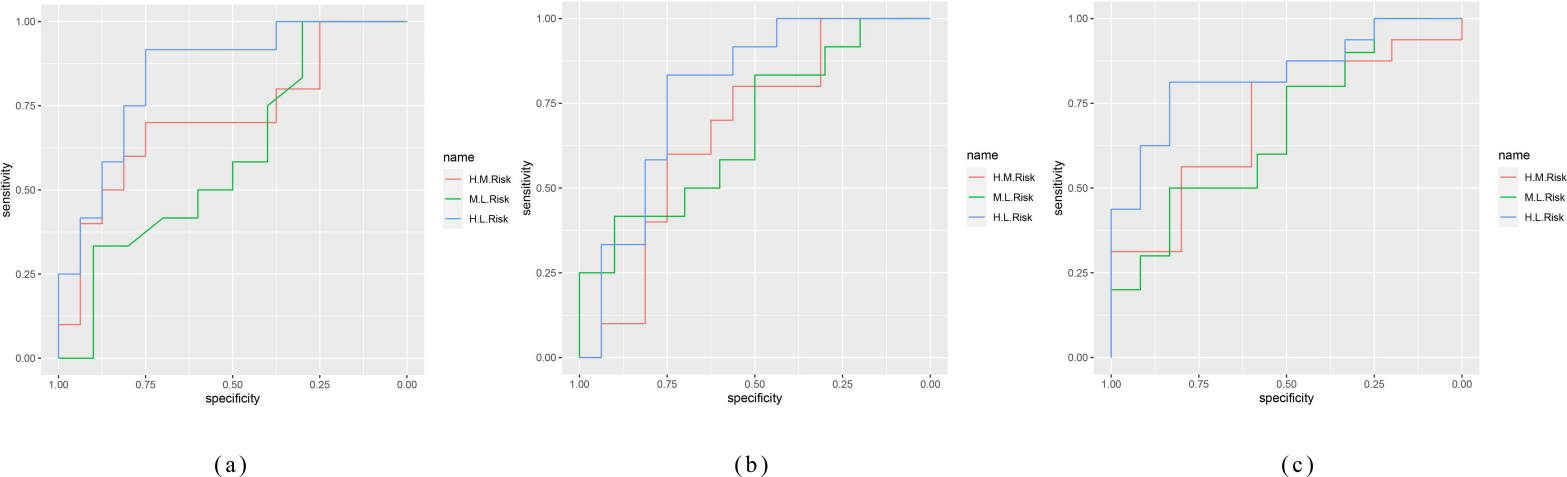
Validation of NLR and PLR as novel biomarkers for neuroblastoma risk assessment using ROC curve analysis. **(a)** ROC curves of NLR for discriminating high- vs. intermediate-risk(blue line), intermediate- vs. low-risk(red line), and high- vs. low-risk(green line) neuroblastoma. **(b)** ROC curves of PLR for the same pairwise comparisons. **(c)** ROC curves of the combination of NLR and PLR (NLR+PLR).

Receiver Operating Characteristic (ROC) analysis was performed to evaluate the capacity of NLR, PLR, and their combination to distinguish high-risk neuroblastoma patients from the low/intermediate-risk cohort. As illustrated in **Figure 2**, both independent markers showed significant diagnostic value. However, the combined NLR-PLR index demonstrated the most robust discriminatory performance, achieving a superior Area Under the Curve (AUC) of 0.833. This synergy suggests that integrating both inflammatory indices provides a more comprehensive reflection of the tumor microenvironment than either marker alone.

#### 3.2.2 Comparative performance of diagnostic indices

The optimal cut-off values for clinical application were determined based on the maximum Youden’s Index.As detailed in **Table 2**, the optimal cutoff for NLR was established at 0.98, while the PLR cutoff was 104.6. Given the limited sample size, we internally validated the model using 1000 bootstrap resamples. All three markers demonstrated the highest AUC values for discriminating high-risk from low-risk patients, with NLR achieving 0.831 (bootstrap-corrected: 0.750), PLR 0.828 (corrected: 0.819), and the combined model 0.833 (corrected: 0.827). The minimal difference between apparent and corrected AUCs for PLR and the combined model indicates good generalizability, whereas NLR showed more pronounced overoptimism (0.831 → 0.750). Both NLR and PLR yielded high sensitivity and specificity in identifying high-risk and low-risk disease, further supporting their potential as reliable, low-cost screening tools in the clinical practice. The bootstrap analysis provided optimism-corrected AUC and calibration metrics. These results demonstrate that the model’s predictive accuracy remains stable and acceptable after internal validation, thereby reinforcing the reliability of our findings.

**Table 2:** NLR and PLR in assessing neuroblastoma risk.

Parameters	Cutoff values	Sensitivity (%)	Specificity (%)	AUC
NLR				
High/Medium-risk	0.975	75	75	0.695
Medium/Low risk	0.480	100	33.3	0.632
High/Low Risk	0.980	75	91.7	0.831
PLR				
High/Medium-risk	135.750	81.8	66.7	0.739
Medium/Low risk	81.835	90.9	41.7	0.682
High/Low Risk	104.675	80	83.3	0.828
NLR+PLR				
High/Medium-risk				0.675
Medium/Low risk				0.706
High/Low Risk				0.833

### 3.3 Violin plots of NLR (a) and PLR (b) stratified by risk level

**Figure 3:**
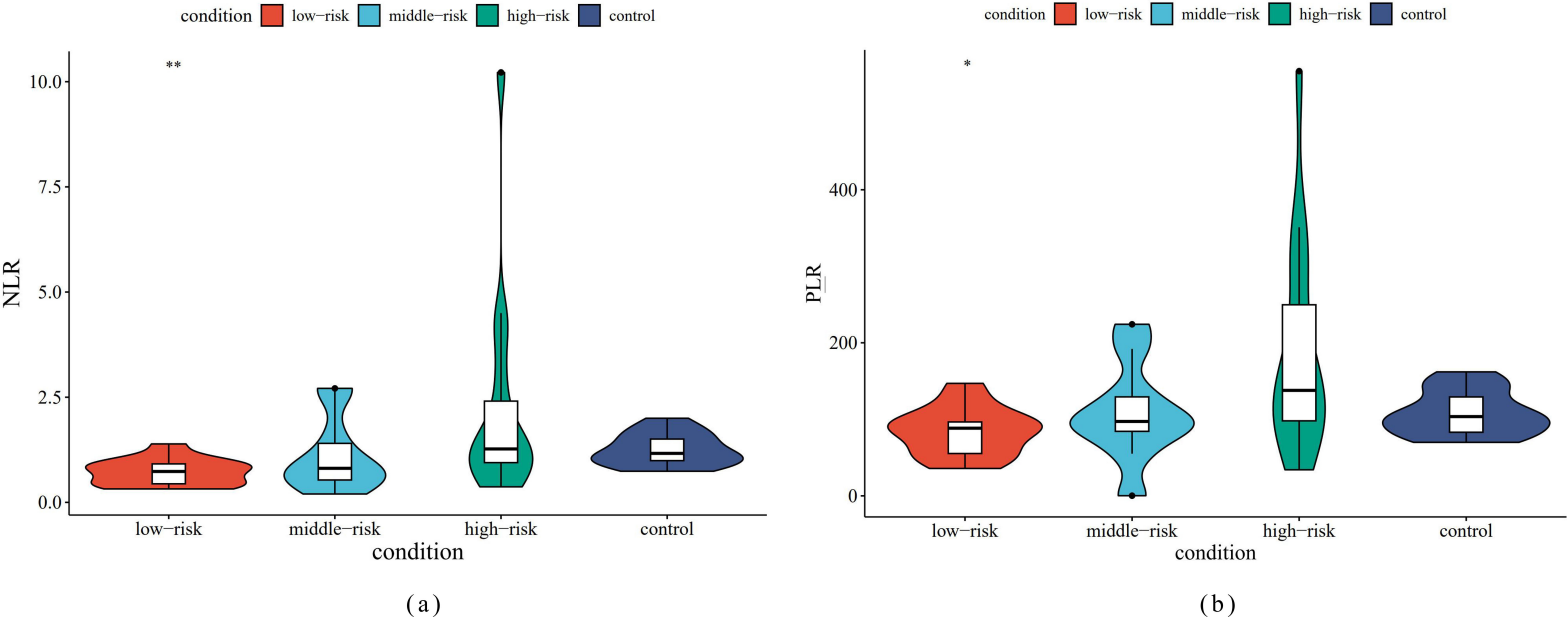
Association of NLR **(a)** and PLR **(b)** with neuroblastoma risk. Distribution of NLR and PLR across risk groups (low, intermediate, and high) and healthy controls.

The violin plots display the density distribution, median (white dot), interquartile range (thick black bar), and 95% confidence interval (thin black line) of each biomarker. As shown in **Figure 3**, both NLR and PLR increased significantly in a stepwise manner with higher risk categories.

### 3.4 Pearson correlation of NLR and PLR with ferritin, LDH and age

As shown in **Table 3**, no significant correlations were detected between any pair of variables (all P > 0.05). Specifically, NLR was not correlated with LDH, ferritin or Age; the same was true for PLR. All correlation coefficients were close to zero, indicating that NLR and PLR vary entirely independently of these direct tumor burden markers. 

**Table 3:** Pearson correlation of NLR and PLR with ferritin, LDH and age.

Variables	P value	Correlation coefficient
NLR-ferritin	0.952	0.010
NLR-LDH	0.649	0.076
PLR-ferritin	0.494	0.114
PLR-LDH	0.547	0.101
NLR-Age	0.139	0.244
PLR-Age	0.144	0.241

Number of Samples 38.

### 3.5 Comprehensive evaluation of model performance: Hosmer–Lemeshow calibration, confusion matrix–based metrics, and DCA analysis

Calibration was assessed by the Hosmer–Lemeshow (H-L) test. A non-significant P value (P>65;0.05)indicates good agreement between predicted probabilities and observed outcomes. The H-L goodness-of-fit test and confusion matrix-derived metrics are summarized in the **Table 4**. For the combined NLR+PLR model, the H-L test yielded chi-square values of 3.21, 3.85, and 1.57 for the High/Medium-risk, Medium/Low-risk, and High/Low-risk comparisons, respectively, with all P values exceeding 0.05 (P = 0.360, 0.278, and 0.666). These results demonstrate that the combined model was well calibrated across all pairwise risk discriminations. In comparison, the individual NLR and PLR models also showed adequate calibration, with H-L P values consistently > 0.05. Calibration plots visually confirmed these findings, showing predicted probabilities closely tracking the ideal line.

**Table 4:** Comprehensive performance evaluation of NLR, PLR, and the combined NLR+PLR model: Hosmer–Lemeshow calibration test and confusion matrix-derived classification metrics across pairwise risk stratum comparisons.

Parameters	H-L test			confusion matrix analysis		
	X^2^	P	Sensitivity (%)	Specificity (%)	AUC	F1 score
NLR						
High/Medium-risk	4.83	0.185	81.2	50	0.763	0.765
Medium/Low risk	6.13	0.105	50	58.3	0.594	0.500
High/Low Risk	1.62	0.655	68.8	66.7	0.750	0.710
PLR						
High/Medium-risk	3.92	0.271	81.2	40	0.756	0.743
Medium/Low risk	2.54	0.468	40	66.7	0.632	0.444
High/Low Risk	4.77	0.189	81.2	83.3	0.819	0.839
NLR+PLR						
High/Medium-risk	3.21	0.360	87.5	60	0.862	0.903
Medium/Low risk	3.85	0.278	60	58.3	0.714	0.700
High/Low Risk	1.57	0.666	87.5	75	0.894	0.839

We derived the optimal cut-off values using the Youden index and constructed confusion matrices. Accuracy, precision (positive predictive value), and F1 score were computed alongside sensitivity and specificity; key metrics are displayed in **Table 4**. The combined NLR+PLR model exhibited superior and robust classification performance: For High/Medium-risk discrimination, it achieved a sensitivity of 87.5%, specificity of 60%, AUC of 0.862, and an F1 score of 0.903, reflecting an excellent balance between precision and recall. For High/Low-risk discrimination, the model attained a sensitivity of 87.5%, specificity of 75%, AUC of 0.894, and an F1 score of 0.839. For Medium/Low-risk discrimination, the sensitivity was 60%, specificity 58.3%, AUC 0.714, and F1 score 0.700. Notably, the combined model markedly outperformed the standalone NLR and PLR markers in nearly all comparisons. For instance, in the clinically more relevant High/Low-risk task, the combined model’s AUC increased from 0.750 (NLR) and 0.819 (PLR) to 0.894, and the F1 score improved substantially. These results reinforce that the combined model not only discriminates well (high AUC) but also makes accurate individual-level predictions, as evidenced by the high F1 score and sensitivity.

**Figure 4:**
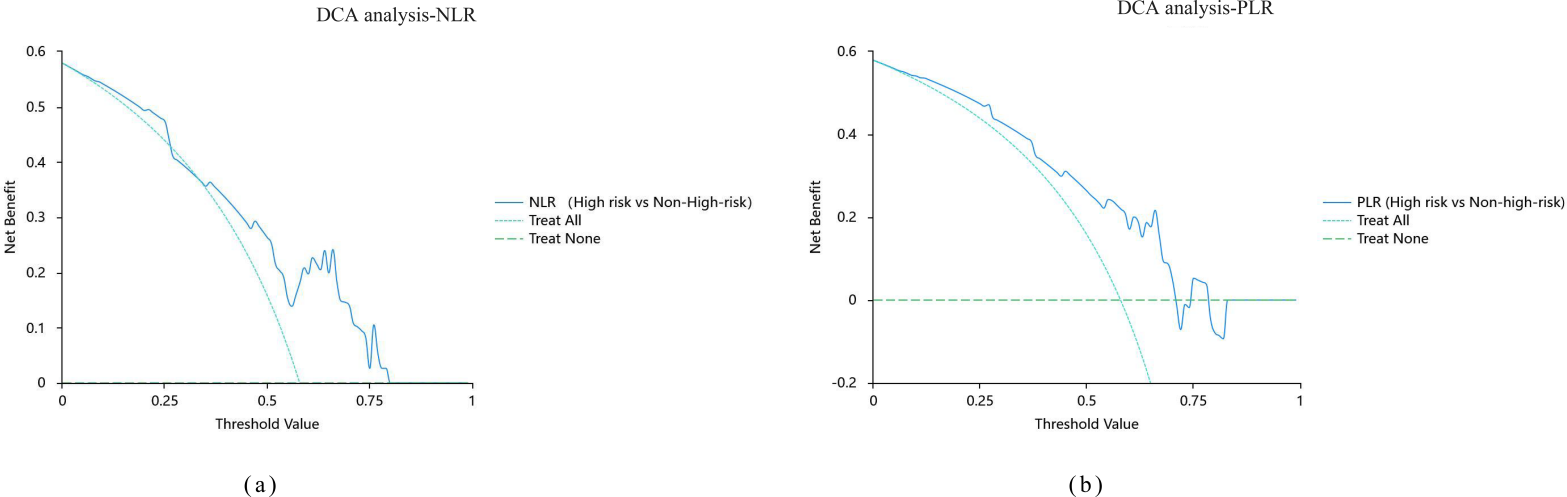
Decision curve analysis assessing the clinical utility of the model in discriminating high-risk from non-high-risk patients **(a, b)**.

To further evaluate the clinical utility of the model, we performed decision curve analysis (DCA) for the discrimination of high-risk versus non-high-risk patients. The analysis shows that our model yields a higher net benefit than the “treat all” or “treat none” strategies within a clinically relevant range, supporting its potential clinical utility.These findings underscore the clinical utility of our model and are detailed in **Figures 4a, b**.

## 4 Discussion

The tumor microenvironment (TME) is increasingly recognized as a site of chronic inflammation, where systemic inflammatory markers derived from routine blood counts offer significant prognostic insight (17–19). In neuroblastoma, the Neutrophil-to-Lymphocyte Ratio (NLR) and Platelet-to-Lymphocyte Ratio (PLR) function as integrated biomarkers that simultaneously quantify systemic inflammation and immune homeostasis. Our findings demonstrate that these composite indices possess superior prognostic capability compared to isolated hematologic parameters, showing a significant association between elevated NLR/PLR values and advanced neuroblastoma risk stratification (P<0.05).

### 4.1 The role of the myeloid and megakaryocytic lineages

Inflammation drives oncogenesis by remodeling the TME with bioactive factors that facilitate tumor progression (20). Neutrophils and platelets play a dual role: they are both participants in this remodeling and quantifiable markers of its severity (21, 22). Neutrophils promote malignancy by secreting tumor-promoting substances—such as reactive oxygen species (ROS), elastase, and prostaglandin E2 (PGE_2_)—which accelerate carcinogenesis, metastatic spread, and angiogenic activation (23). Similarly, platelets regulate the inflammatory response by deploying vasoactive mediators and prothrombotic factors that perpetuate tissue-level immune reactions (24, 25). Through the secretion of VEGF, TGF-β, and PDGF, platelets stimulate angiogenesis and tumor invasion, fostering an increasingly aggressive phenotype (25, 26).The lack of significant correlations between NLR/PLR and LDH/ferritin indicates that these circulating inflammatory cell ratios do not serve as surrogate markers of tumor burden, but rather reflect a distinct host immune response independent of tumor volume and cell turnover. A key biological confounder is catecholamine secretion. Neuroblastomas with high catecholamine output can raise circulating epinephrine and norepinephrine, which activate β-adrenergic receptors on hematopoietic stem cells and bone marrow stroma, driving myeloid mobilization and neutrophilia. This endocrine–paracrine loop may elevate NLR/PLR even without an aggressive tumor–immune interaction. Although our data argue against a purely volume-driven phenomenon, we could not adjust for VMA/HVA; therefore, immune-based cutoff values may overestimate immunological risk in patients with high catecholamine activity and intermediate-risk biology. Prospective studies should measure urinary VMA, HVA, and circulating immune subsets to calibrate NLR/PLR-based stratification according to catecholamine secretion profiles. Until then, the interpretation of elevated NLR/PLR in neuroblastoma should, wherever possible, incorporate a comprehensive assessment of tumor biology that includes catecholamine metabolites.

Future prospective studies are necessary to validate and refine the proposed cut-off points. In particular, we advocate for the routine measurement of urinary VMA and HVA at diagnosis, allowing formal assessment of whether catecholamine secretion independently influences NLR and PLR. Incorporation of VMA/HVA into multivariate prognostic models will help determine whether adjustment of the NLR/PLR thresholds is required for patients with high biological catecholamine activity but otherwise intermediate clinical features, ultimately improving the specificity of these affordable inflammatory biomarkers.

### 4.2 The significance of relative lymphopenia

Conversely, lymphocytes are the primary effectors of the host’s anti-tumor immune response. Tumor-infiltrating lymphocytes exert direct cytotoxic effects on neoplastic cells, forming the biological basis for modern immunotherapy (27). Systemic inflammation in high-risk neuroblastoma is characterized by a “triple threat” profile: neutrophilia, thrombocytosis, and relative lymphopenia (25). During disease progression, lymphocytes often undergo spatial exclusion from the tumor core and functional suppression within the immunosuppressive TME, leading to a progressive depletion of effector cell populations (28).

### 4.3 Risk-based divergence in NLR and PLR

Our data revealed a compelling correlation between neuroblastoma severity and these inflammatory indices. Interestingly, while high-risk patients exhibited NLR and PLR values significantly higher than healthy controls, intermediate- and low-risk patients actually demonstrated *lower* ratios than the control group. The lower NLR and PLR observed in patients with low-risk neuroblastoma may largely reflect the lack of profound systemic metabolic stress and catecholamine secretion characteristic of high-risk disease. In high-risk tumors, catecholamine-driven β-adrenergic stimulation promotes myeloid mobilization from the bone marrow and may concurrently induce lymphocyte apoptosis, thereby raising both NLR and PLR. By contrast, low-risk patients, who harbor localized, metabolically quiescent tumors that do not markedly alter the endocrine milieu, would be expected to maintain a more physiological leukocyte balance. This explanation is more consistent with the available evidence. However, the preservation of a higher lymphocyte count in low-risk patients cannot be entirely dismissed as a permissive cofactor for effective antitumor immunity — a possibility that could be investigated in prospective studies incorporating NLR/PLR along with lymphocyte subset phenotyping.

## 5 Clinical implications

The integration of NLR and PLR into the management of neuroblastoma (NB) offers significant clinical advantages due to their non-invasiveness, cost-efficiency, and universal accessibility (29). While molecular profiling (such as *MYCN* amplification) remains the gold standard, its high cost and technical requirements often limit its availability in resource-constrained settings. In contrast, a routine complete blood count (CBC) is a fraction of the cost—often less than one-tenth that of genetic testing—and provides actionable data with immediate turnaround times.

### 5.1 A novel, integrated risk stratification model

Our findings suggest that these inflammatory biomarkers could potentially refine the existing International Neuroblastoma Risk Group (INRG) staging system. By incorporating NLR and PLR, clinicians may better address the clinical heterogeneity of “intermediate-risk” cohorts. We propose a risk-adapted therapeutic framework based on these indices:

- High-Risk Profiles (Elevated NLR >0.98/PLR >104.6): For patients traditionally classified as intermediate-risk who exhibit elevated inflammatory markers, we recommend intensified surveillance. This includes more frequent imaging, monthly monitoring of serum LDH and urinary catecholamines (HVA/VMA), and a lower threshold for MIBG scan evaluation. For established high-risk patients with concurrent NLR/PLR elevation, these markers may support the case for therapeutic intensification, such as anti-GD2 immunotherapy consolidation post-induction and autologous hematopoietic stem cell transplantation (ASCT).
- Discordant Low-Inflammation Profiles: Conversely, patients with high-stage disease but low inflammatory indices could be candidates for treatment de-escalation. This approach may mitigate therapy-related toxicities in chemotherapy-intolerant patients or those recovering from severe infections.

### 5.2 Integration into standard diagnostic workflows

We propose that NLR and PLR be incorporated into standard prognostic workflows. Patients exceeding the identified cutoffs (NLR >0.98; PLR >104.6) at diagnosis should be considered for risk reclassification. Clarifying the role of these readily accessible biomarkers could help oncologists worldwide to stratify risk early and with greater precision, thereby facilitating personalized interventions that may ultimately improve patient survival.

Refined Limitations and Conclusions

## 6 Limitations

While this study provides compelling evidence for the utility of systemic inflammatory markers, it has several limitations. First, the retrospective, single-center design may introduce selection bias, and the findings may not generalize to other populations with different demographic or clinical characteristics.Second, the sample size (n=38), while statistically significant, is modest; The small sample limits statistical power, particularly for subgroup analyses (e.g.stratifying by MYCN status or age), which we were unable to perform reliably. Consequently, our reported cut-off values for NLR and PLR should be viewed as exploratory; they may be unstable and are likely influenced by sampling variability.therefore, these findings should be viewed as a foundational pilot for larger investigations. Third, we could not adjust for potential confounders such as stage, MYCN, and VMA/HVA status due to incomplete/unavailable data. Therefore, we cannot determine whether the observed associations are independent or confounded by these established prognostic factors. Future prospective studies with multivariate adjustment(eg.VMA/HVA) are needed to test for independence. Finally, although we excluded patients with active infections, other subclinical inflammatory states could potentially influence NLR and PLR values.Given these limitations, our results should be considered hypothesis-generating rather than definitive. Prospective, multi-center studies with larger cohorts and longitudinal follow-up are required to validate these cutoff values and assess their impact on overall and event-free survival.

## 7 Conclusions

In conclusion, pretreatment NLR and PLR are robust, cost-effective, and easily accessible biomarkers that significantly correlate with neuroblastoma risk stratification. Our data demonstrate that elevated NLR and PLR values are indicative of high-risk disease and more aggressive clinical phenotypes. Notably, the combined NLR-PLR index provides superior diagnostic accuracy compared to either marker alone, with an AUC of 0.833.

By establishing optimal cutoff values of 0.98 for NLR and 104.6 for PLR, this study offers a practical framework for clinicians to enhance risk prediction, particularly in resource-limited settings. We recommend prospective studies with extended follow-up, mandatory collection of OS and EFS, and inclusion of MYCN status, age, LDH, ferritin, and VMA/HVA to validate and refine the proposed NLR/PLR cutoffs. While large-scale prospective trials are necessary to establish Level I evidence, our findings support the integration of these inflammatory indices into standard neuroblastoma prognostic workflows to facilitate more precise, risk-adapted therapeutic strategies.

## Data Availability

The raw data supporting the conclusions of this article will be made available by the authors, without undue reservation.
